# The price behavior characteristics of China and Europe carbon emission trading market based on the perspective of time scaling and expected returns

**DOI:** 10.1371/journal.pone.0298265

**Published:** 2024-02-14

**Authors:** Peng-Cheng Zhang, Jie Cheng

**Affiliations:** Shandong Institute of Petroleum and Chemical Technology, Dongying, China; National Technical University of Athens: Ethniko Metsobio Polytechneio, GREECE

## Abstract

China has the world’s largest carbon market in terms of greenhouse gas emissions, but its system needs to be improved and enhanced. In comparison, the European carbon market stands as the most mature and well-developed carbon market globally. Carbon trading prices, serving as a barometer for the carbon market, are significantly influenced by investor behavior. Therefore, it is necessary to analyze the characteristics of carbon trading prices in both China and Europe, considering the impact of investor trading intervals and psychological expected returns. This study utilizes the Zipf method to characterize the dynamic behavior of carbon trading prices between China and Europe, conducting a comparative analysis. The results show distinctive asymmetry in the behavior of carbon trading prices in both markets. In the Chinese market, when τ < 277, the absolute deviation *d*_*a*_(*τ*, *ε*) value gradually changes but consistently indicates a bullish trend. However, when τ ≥ 277, the *d*_*a*_(*τ*, *ε*) value surges rapidly, reflecting a pronounced bullish sentiment among investors toward carbon trading prices in China. In the European market, within the sample period, regardless of variations in τ and ε, the *d*_*a*_(*τ*, *ε*) value shows a linear upward trend, indicating a significant overall bullishness in prices. This suggests a higher probability of long-term bullishness in carbon trading prices. Investors’ investment time scale (τ) and expected returns (ε) both influence the behavior of carbon trading prices in both China and Europe. Generally, a longer τ implies a higher probability of bullishness. As for ε, higher values lead to more extreme judgments on price movements, resulting in greater distortion in carbon trading prices. Short-term investors (τ<1 month) anticipate extreme fluctuations, exhibiting random behavior when ε < 0.15 and converging rapidly to extreme values of 1 or 0 when ε ≥ 0.15. Long-term investors (τ>quarter) are less biased, expressing a bullish outlook on both Chinese and European carbon prices. With increasing ε, the probability of bullishness either increases or decreases rapidly until reaching the saturation point. Once saturated, there is no further distortion in carbon price behavior. Furthermore, the Chinese carbon market displays a positive trend in carbon trading prices and a higher probability of long-term bullishness. For the European market, lower expected returns contribute to considerable carbon trading price fluctuations, exacerbating risk and uncertainty. The results of this study contribute to understanding the diverse trading behaviors in Chinese and European carbon markets and provide guidance for avoiding extreme volatility in carbon trading prices.

## Introduction

The global economic development is confronted with escalating resource and environmental constraints, with a heightened focus on carbon emissions as a critical issue.. Urgent measures are necessary to propel a low-carbon transformation, foster carbon reduction, and advance the development of clean energy. An important instrument in reducing emissions is the carbon emissions trading system. As global industrialization accelerates, carbon emissions have surged from 24.15 billion tons in 1998 to 36.3 billion tons in 2021. Due to the imperfect energy structure, global temperatures may continue to rise for the next 20 years.

Currently, there are 31 carbon trading markets and 30 carbon tax mechanisms globally, covering carbon emissions equivalent to nearly 12 billion tons of carbon dioxide equivalent in 46 countries and 32 regions. This constitutes approximately 22% of the total global greenhouse gas emissions. By the year 2022, 28 carbon emissions trading systems were operational globally, and the proportion of emissions covered by the carbon market system had increased from less than 8% in 2014 to 17%.

Since the establishment of the world’s first carbon emissions trading market by the EU in 2005, 32 carbon markets have been operational in 38 national jurisdictions and 31 sub-national jurisdictions globally. These markets cover about 1 billion tons of greenhouse gas emissions, accounting for 17.0% of global greenhouse gas emissions. From a regional perspective, carbon markets in developed regions developed earlier. Presently, the US and Europe occupy a dominant position in the global carbon trading market, particularly in terms of trading volume. Compared with the US, Europe’s carbon market is more mature and complete. Key players in the carbon emissions trading landscape include the European Climate Exchange (ECX), the Nordic Power Exchange (NP), the BlueNext exchange in France, the European Energy Exchange (EEX), and the French Future Power Exchange (Powernext). Among them, the European Climate Exchange has the largest trading volume, and the market share of carbon financial contracts exceeds 80% of the total trading volume of carbon financial products in Europe.

In East Asia, China, South Korea, and Japan have each established their national and regional carbon markets. In 2011, aligning with the 12th Five-Year Plan for National Economic and Social Development of the People’s Republic of China (2011–2015), which advocated for the "gradual establishment of a carbon emissions trading market," China launched pilot programs in Beijing, Tianjin, Shanghai, Chongqing, Hubei, Guangdong, and Shenzhen. Expanding on this initiative, Fujian Province became the eighth carbon trading pilot in 2016. On July 16, 2021, China officially launched and operationalized its national carbon emissions trading market. This marked a crucial development, with the power generation industry being the pioneering sector included in the national carbon market, which included more than 2,000 key emitting units.

China’s carbon market is poised to emerge as the world’s largest in terms of greenhouse gas emissions coverage. However, compared with developed regions such as Europe and North America, the developmental framework of China’s carbon market requires ongoing refinement and enhancement. Carbon prices reflect the operation of the carbon market and are of great significance to improving the carbon market. Therefore, this study focuses on the price fluctuations of carbon trading in China and Europe, compares the carbon trading prices between China and Europe, and proposes recommendations for high-quality development of the carbon market.

In light of the carbon neutrality proposal, numerous scholars have shifted their focus toward the carbon trading market. Their primary focus lies in understanding the carbon trading market, delineating its developmental path, evaluating carbon market efficiency, analyzing trends in carbon price volatility, assessing carbon market risks and factors influencing the carbon market, and proposing strategies for the construction of the carbon market. Their collective efforts have yielded significant contributions to the advancement of the global carbon market.

In terms of climate change and carbon dioxide emissions reduction. Sariannidis, N, et al. (2013) [1] found a negative correlation between the performance of socially responsible firms and the increase in global CO2 emissions. Zerva, A. et al. (2018) [2] proposed that effective citizen participation in emission reduction necessitates increased governmental involvement and substantial improvements in areas such as industry and business pollution reduction, civil protection, information provision, public awareness, and education on climate change. Zafeiriou, E et al. (2018) [3] proposed that the asymmetric impact of agricultural income on carbon emissions offers policymakers tools for promoting both increased agricultural income and successful mitigation of greenhouse gas effects.

Kathakalis, C, et al (2019) [4] quantified the relationship between crude oil prices, sustainable economic growth, and environmental policies on a global scale. Ioanna, N. et al (2022) [5] classified SHs as low, medium, or high priority based on a priority index value. At the national level, ministerial directorates were classified as high priority, followed by academic and research centers, which were classified as medium priority. Sebos, I. et al. (2023) [6] identified the lack of human and financial resources, particularly among public institutions, as the main obstacle to addressing and adapting to climate change in terms of public policy planning in Greece.

Kyriakopoulos, G. L. et al. (2023) [7] found that the Greek national strategic framework is consistent with the objectives and priorities of European policies regarding the environment and the climate. However, targeted actions are needed to manage the Greek agricultural sector to properly address the risks of climate change, environmental degradation, and the excessive use of natural resources. Ioannis Sebos et al. (2023) [8] scrutinized principal contributors to emissions, including enteric fermentation, manure management, rice cultivation, and agricultural soils. Losada-Puente, L. et al. (2023) [9] showed that the harmonization of renewable energy legislation in Southern Europe remains incomplete, with notable differences in implementation between countries.

Kyriakopoulos GL. (2022) [10] outlined main research dimensions, including environmental sustainability, reduction of greenhouse gas emissions, optimal exploitation of RES, social awareness, increased energy efficiency, and pollution relief in urban/built environments. Papadogiannaki, S.et al. (2023) [11] suggested that adopting measures post-COVID-19 could significantly decrease greenhouse gas emissions. Progiou, A. G. (2022) [12] attributed considerable reductions in air pollutant emissions and improved air quality levels to lockdown restrictions during the COVID-19 pandemic. Kyriakopoulos, G. L. (2023) [13] addressed the key motives and challenges related to joint adaptation and mitigation actions. Akkermans, S. (2023) [14] proposed four mitigation pathways for Tajikistan, each incorporating different policy intensity levels. Bozoudis, V. (2022) [15] studied carbon emissions from medical activities.

In terms of understanding and developing the carbon trading market, Xiaohong Chen et al. (2013) [16] analyzed the price mechanism of the European Union Emissions Trading System (EU ETS) using the EGARCH (1,1) model, indicating significant differences in price mechanisms and volatility between the first and second phases. Dmitry Fedosov (2017) [17] explored connecting emission trading systems with data sources beyond traditional academic sources, vital for the development of connected carbon markets. Liang Yu (2019) [18] drew lessons from the EU carbon trading mechanism, offering insights into the construction of the Chinese carbon market through the study of the linkage between the EU carbon market and the high-carbon company stock market. Wang Yong et al. (2019) [19] used an improved CGE model to evaluate the impact of energy structure changes on the macroeconomy and carbon market, providing a reference for China’s future energy structure adjustments. Zhang Zhong Xiang (2022) [20] proposes specific areas for regional carbon markets to actively enhance the synergy effects with the national carbon market. Xiang Yue et al. (2023) [21] established a multi-objective investment evaluation method for low-carbon power systems, providing pathways and assistance for low-carbon development based on multiple development goals such as energy security, ecological protection, economic development, and technological progress.

In the realm of carbon market efficiency, Luiza Maia de Castro et al. (2013) [22] simulated the Brazilian carbon market model, highlighting the importance of market mechanism efficiency and political processes in determining emission allowance allocation standards. George Daskalakis (2013) [23] used simple technical analysis rules and predictions to evaluate the profitability of trading strategies, confirming the gradual maturity of the European carbon market. Zhang Shiyi et al. (2020) [24] reviewed the trading performance in eight regional carbon markets in China, employing robust variance ratios (VR) tests to assess efficiency at different periods. They proposed suggestions for enhancing the efficiency and maturity of China’s carbon market, including strengthening legislation, refining market design, and constructing information platforms. Xianfeng Liu et al. (2020) [25] measured the efficiency of seven carbon markets, using variance ratio tests and detrended fluctuation analysis, demonstrating that the fractal market hypothesis can effectively measure carbon market efficiency compared to the efficient market hypothesis. Chen Yingqi et al. (2021) [26] conducted a wild Bootstrap variance ratio test on market efficiency using the latest and comprehensive data from the Hubei pilot carbon market, providing new evidence about the efficiency of China’s carbon market pilot. Zafeiriou, E et al. (2014) [27] observed a positive correlation between emissions or gasoline prices and ethanol prices while noting an inverse relationship in the case of crude oil.

Regarding carbon price volatility trends, Kaile Zhou et al. (2018) [28] used the vector error correction (VEC) model to explore the dynamic relationships among energy prices, macroeconomic indicators, air quality, and carbon emission trading prices. They also analyzed carbon price volatility characteristics using the generalized autoregressive conditional heteroskedasticity (GARCH) model. Hao Wu et al. (2020) [29] focused on the main factors influencing carbon prices. They established quantitative models using static panel models (FGLS) and dynamic panel models (PVAR) across various pilot trades from 2014–2018, generating valuable insights into the driving factors of carbon prices in China. Xu Hua et al. (2021) [30] selected five forecasting models as benchmark models and conducted empirical analysis using price data from both the EU and China’s seven pilot carbon markets from June 19, 2014, to October 9, 2020. Their research delved into carbon price volatility trends and offered suggestions for improving China’s pilot carbon market. Li Jingyu et al. (2022) [31] studied Chinese carbon price volatility using adaptive Fourier decomposition (AFD). Chen Fan et al. (2023) [32] introduced a novel dual quantile regression model that reflects the volatility characteristics of carbon prices, focusing on the impact of the Chinese stock market and the EU carbon market on the prices of the Chinese carbon market under diverse market conditions. They concluded that, to some extent, international carbon markets and stock risks can influence the Chinese carbon market, proposing relevant countermeasures and suggestions. Rudnik et al. (2023) [33] analyzed a wide range of factors affecting EUA price conditions. They compared results from various feature selection methods and modeled the influence of key determinants of CO2 price daily forecasts using fuzzy rules, thereby obtaining additional information about the changing trends in EUA price levels. Li, D.et al. (2023) [34] proposed the MEPT-ICEEMDAN-CTCN model, showcasing significant superiority in multi-step-ahead and quantile forecast. Känzig, D. R. (2023) [35] leveraged institutional features of the European carbon market and high-frequency data. The study documented that a more stringent carbon pricing regime leads to higher energy prices, lower emissions, and more green innovation.

Regarding carbon market risks, Zhen-Hua Feng et al. (2012) [36] measured the value at risk (VaR) of the EU ETS spot and futures markets using extreme value theory and the GARCH model. Linda Meleo (2014) [37] identified key factors that qualitatively measure "competitiveness risks" related to the EU-ETS and evaluated both actual and potential risks affecting the sector. Meleo’s work served as a foundational starting point for subsequent research in Italy and Europe, as well as for future policy formulation. Balcılar Mehmet et al. (2015) [38] employed various methods to investigate the risk spillover between energy futures prices and European carbon futures contracts. Lei Jiao et al. (2018) [39] developed an economic state dependence (SD) method to assess carbon market risk through the value at risk (VaR), incorporating macroeconomic fundamentals into carbon return VaR modeling and forecasting. Bangzhu Zhu et al. (2019) [40] proposed a multiscale VAR method based on empirical mode decomposition (EMD) to comprehensively understand the volatility characteristics of carbon market risks, providing new insights for exploring the evolution of European carbon market risks. Yang Xianzi et al. (2020) [41] developed a combined model for measuring the risk of the Chinese carbon market, providing a basis for the description theory and methodology of carbon price specificity and improving the accuracy of carbon market risk measurement. Their research offered a novel perspective for studying the evolution of China’s carbon market risks. Li Guanxu (2022) [42] conducted a measurement study on the volatility risk of China’s carbon trading market, exploring aspects such as risk volatility persistence, volatility trends, return volatility magnitude, and VaR volatility degree. The findings offer effective suggestions for China’s carbon market trading. Vellachami Sanggetha et al. (2023) [43] examined the volatility transmission from the European energy market to the carbon market, enhancing understanding of information and risk overflow mechanisms from the energy market to the carbon market and serving as a basis for policy-making, risk management, and portfolio optimization decisions.

In terms of the factors affecting the carbon market, Steinar Andresen et al. (2015) [44] examined the potential effectiveness of the Paris Agreement on EU climate policies and the carbon market, affirming the impact of policies on the development of the European carbon market. Wang Yong et al. (2019) [19] used an improved CGE model to evaluate the impact of changes in energy structure on the macroeconomy and carbon market, providing references for China’s future energy structure adjustment. Zhao Zhixuan (2022) [45] analyzed the dynamic interaction mechanism between the carbon emission quota transaction price in Guangdong Province and factors such as economic development level, foreign carbon price, and new energy price, using a vector autoregression (VAR) model. The study found that carbon pricing plays an important role in the development of a low-carbon economy and is a significant factor influencing the effective operation of the carbon market. Zhang Xu et al. (2023) [46] established a VAR model using daily data on investor attention and domestic and foreign carbon trading indexes, confirming that investor attention also affects the returns of the carbon trading market.

Regarding carbon market development, Jing Ma et al. (2010) [47] provided an overview of the carbon market and analyzed its development prospects, emphasizing the necessity of carbon market construction in China and proposing a technical roadmap for its development. Franziska Wolff (2011) [48] presented a conceptual framework for developing a regulated global carbon market. Yifei Hua et al. (2019) [49] gave an overview of the operation and development of eight carbon market pilots in China, highlighted existing problems in the national carbon market, discussed elements that need improvement during the establishment process, and explored the feasibility of connecting future carbon markets in China. Xia Yingzhe et al. (2022) [50] analyzed the current status, problems, and countermeasures of the construction of a national carbon market in China. Sattarhoff et al. (2022) [51] calculated the information efficiency of the European carbon market using a new method based on intermittency coefficients. Wangle Zexiang et al. (2023) [52] developed an institutional flexibility framework to study how the established norms and institutional constellations of the EU legislative trinity contribute to mitigating the impact of expanding designated ETS policies, providing a new perspective for assessing the dynamic performance of the ETS.

In summary, existing literature extensively covers topics such as carbon price volatility trends, carbon market risks, factors influencing the carbon market, carbon market efficiency, and carbon market development, providing important references for examination of both domestic and international carbon markets. However, research on carbon price time scale, and expected returns remains relatively limited. Despite China being the world’s largest carbon market in terms of greenhouse gas emissions, its system requires improvement and enhancement. Conversely, the European carbon market stands out as the most mature and well-developed globally.

In light of this context, this study centers on China’s carbon market and the European carbon market, investigating the impact of investor trading intervals and psychological expected returns on investment decisions and exploring the influence of different investment scales and expected returns on the price volatility behavior of carbon markets. This research aims to provide comprehensive information support and decision-making reference for investors, serving as a foundational resource for global carbon market price policy formulation.

## Methods

### Research methods

#### Zipf method

The Zipf analysis was proposed by American linguist G.K. Zipf in the 1930s, who found that the frequency of each word in a longer article was counted, in order of decreasing frequency. Let f represent the word frequency. If each word is assigned a rank R with the rank of the most frequent word designated as 1, the following power law can be observed:

$$R∼R^{-\xi }$$

For arbitrary languages, the exponential *ξ* is close to 1.

Zipf analysis has been applied in the field of market analysis in recent years, particularly in mapping the up and down information within the price time series. This technique typically involves encoding two characters (such as "1" and "-1" or "u" and "d"), creating a new string sequence. Then, the implied information from this generated sequence is used to analyze the dynamic behavior of the original sequence. If the dynamic behavior follows a random walk, the probabilities of occurrence for "1" and "-1" should be equal, resulting in *ξ* being equal to 0. Conversely, if the probabilities of occurrence for "1" and "-1" are not equal, it indicates that the dynamic behavior exhibits trend and long-term memory characteristics.

By applying Zipf analysis to map the actual price time series of China and Europe’s carbon markets into character sequences and analyzing the probabilities of rise and fall at different time scales, the information embedded in price fluctuations can be combined with speculators’ investment time scale and investor expectations to accurately analyze the dynamic behavior of carbon market prices.

Let P(t) = {p(t_1_), p(t_2_),⋯,p(t_n_)}, p(t_i_) = p_i_(i = 1,2,⋯,n) be the carbon trading price time series, r_i_(τ) represents the return rate on the i-th day given the investment time scale τ, then:

$${\text{r}}_{\text{i}}\left(\tau \right)=\frac{\text{p}\left({\text{t}}_{\text{i}}+\tau \right)-\text{p}\left({\text{t}}_{\text{i}}\right)}{\text{p}\left({\text{t}}_{\text{i}}\right)},\text{i}=1,2,\cdots ,\text{n}-\tau$$ (1)

We obtain the return rate sequence r(τ) = {r_1_(τ), r_2_(τ),⋯, r_n−τ_(τ)} in carbon trading τ. Then, we map r(τ) to {1, 0, -1} using Eq (2):

$$f_{i}\left(\tau ,\varepsilon \right)=\left\{\begin{array}{c} 1,r_{i}\geq \varepsilon \\ 0,-\varepsilon <r_{i}<\varepsilon \\ -1,r_{i}\leq -\varepsilon \end{array}\right.$$ (2)

Thus, we generate a new sequence f(τ,ε) = {f_1_(τ,ε), f_2_(τ,ε),⋯,f_n−τ_(τ,ε)} that contains fundamental information about price fluctuations. Specifically, we consider τ = 1,5,22,64,125,250 as the reference time scale, corresponding to 1 trading day, as well as 1 week, 1 month, 1 quarter, 6 months, and 1 year trading days. ε represents the threshold of investors’ expected returns, with "1", "0", and "-1" indicating price increase, no change, and price decrease in carbon trading, respectively.

Based on this, we examine the frequency of carbon trading price changes influenced by parameters τ and ε. Let *n*_+_(*τ*, *ε*), *n*_0_(*τ*, *ε*) and *n*_−_(*τ*, *ε*) represent the number of occurrences of price increases (1), no change (0), and price decreases (-1) in the sequence *f*(*τ*, *ε*), satisfying *n*_+_(*τ*, *ε*) + *n*_0_(*τ*, *ε*) + *n*_−_(*τ*, *ε*) = *n* − *τ*. We define the absolute change frequency of carbon trading prices for increase (1), no change (0), and decrease (-1) as Eq (3):

$$\begin{array}{l} {\text{p}}_{+}\left(\tau ,\varepsilon \right)={\text{n}}_{+}\left(\tau ,\varepsilon \right)/\left(\text{n}-\tau \right)\\ {\text{p}}_{0}\left(\tau ,\varepsilon \right)={\text{n}}_{0}\left(\tau ,\varepsilon \right)/\left(\text{n}-\tau \right)\\ {\text{p}}_{-}\left(\tau ,\varepsilon \right)={\text{n}}_{-}\left(\tau ,\varepsilon \right)/\left(\text{n}-\tau \right) \end{array}$$ (3)

The relative change frequency is defined as shown in Eq (4):

$$\begin{array}{l} \phi_{+}\left(\tau ,\varepsilon \right)=n_{+}\left(\tau ,\varepsilon \right)/\left(n_{+}\left(\tau ,\varepsilon \right)+n_{-}\left(\tau ,\varepsilon \right)\right)\\ \phi_{-}\left(\tau ,\varepsilon \right)=n_{-}\left(\tau ,\varepsilon \right)/\left(n_{+}\left(\tau ,\varepsilon \right)+n_{-}\left(\tau ,\varepsilon \right)\right)\; \end{array}$$ (4)

Similarly, the absolute deviation, which is the difference between the absolute change frequencies of price increases (1) and price decreases (-1) in carbon trading, is expressed using Eq (5):

$$d_{a}\left(\tau ,\varepsilon \right)=p_{+}\left(\tau ,\varepsilon \right)-p_{-}\left(\tau ,\varepsilon \right)$$ (5)

#### Economic significance of parameters τ and ε and model assumptions

With the growing demand for carbon emission quotas, the carbon trading market garnered increased attention from investors. The investment behavior and psychological expectations of investors play a pivotal role in influencing the dynamics of the carbon trading market. When investors choose different trading time intervals, their expected returns are not consistent.

In light of this, the article introduces parameters τ and ε. τ represents the investment time scale of carbon trading investors, and a higher value of τ indicates longer trading time intervals. ε(ε > 0) represents the threshold of investors’ psychological expected returns, namely the maximum expected value of profits (ε) and the maximum risk tolerance value (−ε). For investors, their subjective assumption of the threshold of psychological expected returns (ε) determines whether the carbon trading price rises or falls.

Given the inherent uncertainties in the carbon trading market, such as uncontrollable risks and uncertain trading costs, investors respond to various scenarios. When the return rate exceeds a certain threshold (r_i_ ≥ ε), investors may perceive that the carbon trading price has reached their expected return and choose to sell their carbon trading assets to secure differential income. When considering transaction costs, if the current carbon trading price is perceived as relatively stable, investors may opt to retain their assets, anticipating a more favorable opportunity (−ε < r_i_ < ε). However, if the carbon trading price falls significantly beyond their risk tolerance (r_i_ ≤ ε), some investors may choose to purchase and retain assets, with the expectation of selling at a higher price when the carbon trading price rebounds, thereby maximizing profits. Therefore, ε serves as an indicator of investors’ psychological tolerance for fluctuations in carbon trading prices, reflecting their optimism towards the carbon trading market and, to some extent, indicating their risk propensity.

#### Data description

The national carbon emission quota (CEA) trading price published by the Shanghai Environment and Energy Exchange was selected for China’s carbon market. The sample period spanned from July 16, 2021, to August 23, 2023, consisting of 512 observations. For the European carbon market, the trading prices of ECX-EUA from the European Climate Exchange were used, covering the period from August 6, 2021, to August 23, 2023, also comprising 512 observations. Through the application of Eqs (2) to (5), preprocessing was conducted on the carbon emission trading prices for both China and Europe, resulting in the carbon trading price series (τ), return rate series, price change series for different values of τ and ε, and absolute and relative change frequency series, as well as the absolute deviation series.

## Results and discussion

### The impact of parameters τ and ε on the price behavior of carbon trading between China and Europe

**Fig 1:**
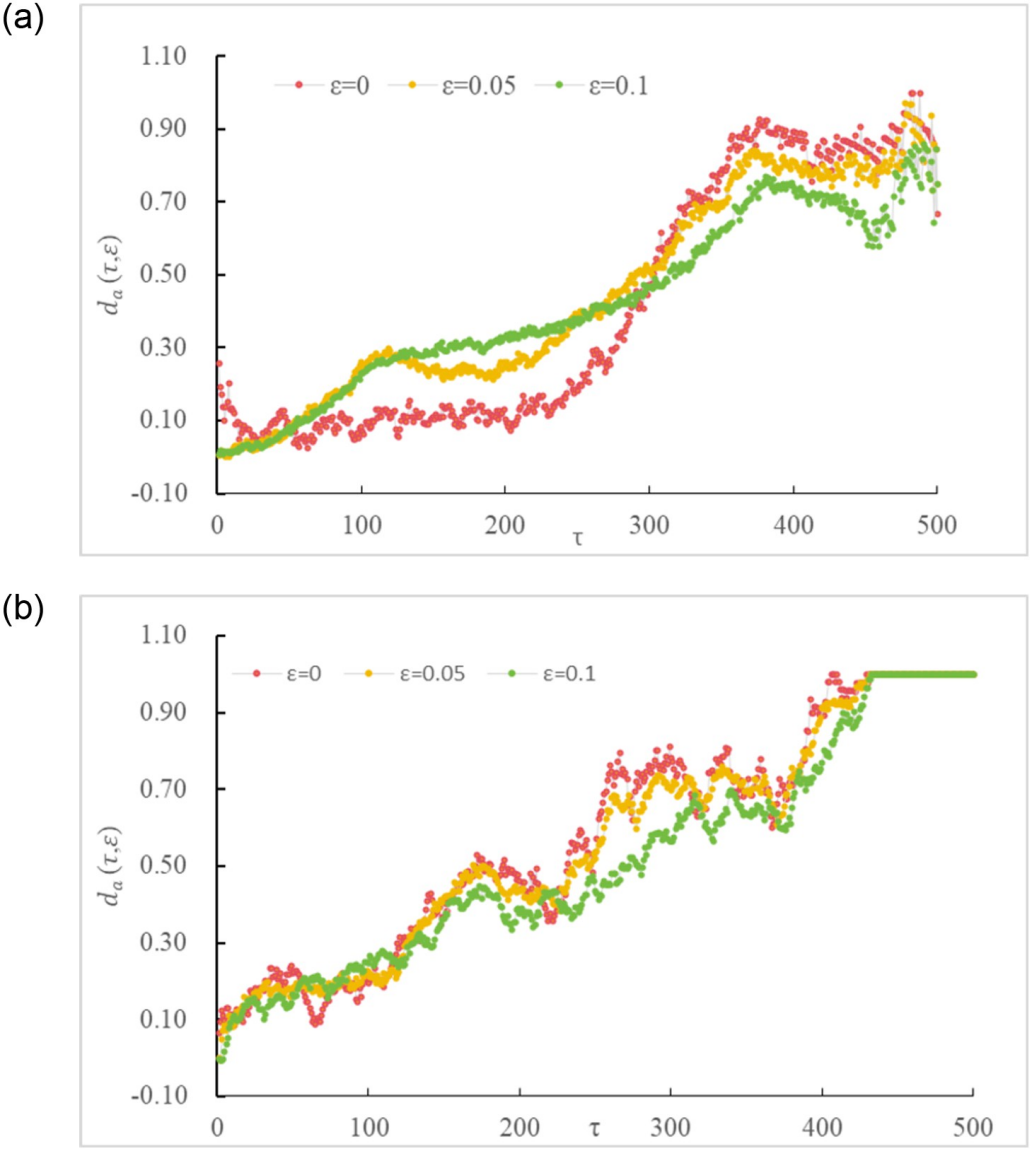
Absolute deviation of the bullish/bearish frequency of carbon trading prices under various time scales. (a) China 1 ≤ τ ≤ 500 and (b) Europe 1 ≤ τ ≤ 500.

Based on Eq (2), an absolute deviation analysis was conducted on the price change series (f_i_(τ, ε)) of carbon trading between China and Europe under different investment time scales (τ) and expected returns (ε). The results are shown in Fig 1, indicating that various investment time scales (τ) and expected returns (ε) exert certain effects on carbon trading prices.

When only considering trading time interval (τ) without factoring in investors’ expected returns (i.e., ε = 0), the following observations can be made:

1. The absolute deviations under different τ values are not equal to 0, indicating an impact on the price fluctuations of carbon trading between China and Europe.
2. Carbon trading prices in China and Europe exhibit asymmetric behavior. Even in extreme cases of ε = 0 and τ = 1, the absolute deviations are not 0 but 0.256 and 0.065, respectively, both greater than 0.
3. For the Chinese market, as τ extends, investors are more bullish on the carbon trading prices in China. This is attributed to the smooth operation of the carbon trading prices in China, with prices consistently rising. When τ < 277, the value of d_a_ increases from 0 to 0.3, indicating a gradually increasing bullish sentiment. When τ ≥ 277, d_a_ sharply increases from 0.3 to 1, signaling a significantly bullish sentiment among investors. This is mainly due to the official launch and operation of the carbon emission trading market in China, which has become the largest carbon market globally in terms of greenhouse gas emission coverage. The Chinese government actively explores carbon trading development, implementing "dual-carbon" goals.
4. In the European market, due to stringent carbon neutrality goals and long-term supply shortages, Europe has established a mature price system. Investors may benefit to varying degrees during this period, leading to a linear upward trend in d_a_, indicating an overall bullish sentiment towards prices.

When considering the factor of investors’ psychological expectations (i.e.,ε > 0), with the change of τ, the following observations can be made:

1. ε influences the price fluctuations of carbon trading between China and Europe to a certain extent, as the absolute deviations under different ε values are greater than 0.
2. Under different τ values, various ε values (such as ε = 0.05 and ε = 0.1) result in different absolute deviations. However, the overall trend of absolute deviations remains relatively stable, suggesting that ε may distort the behavior of carbon trading prices in China and Europe, driving changes in carbon trading prices.

### Parameter ε and the classification of investor types in the China-Europe carbon trading market

**Fig 2:**
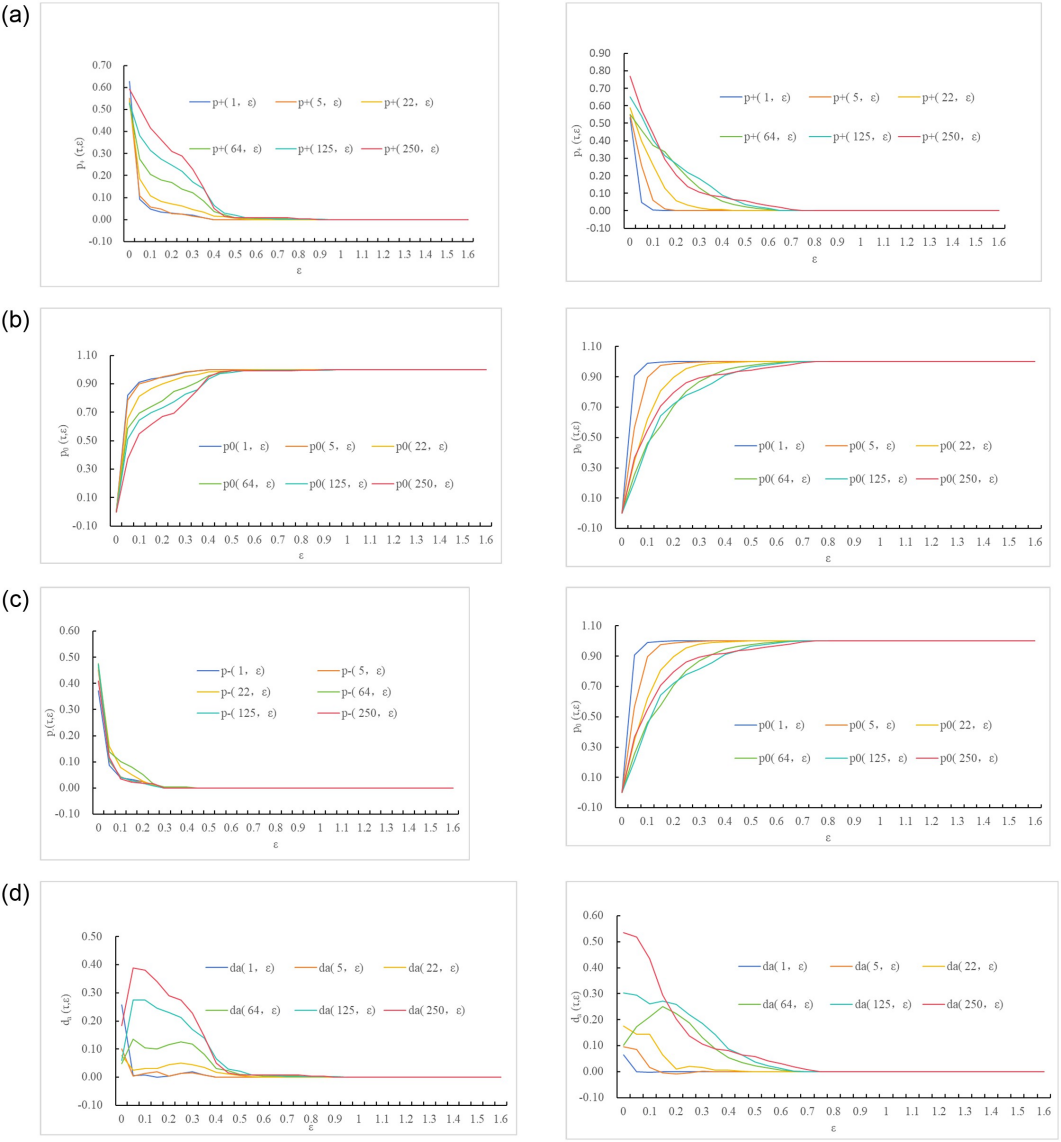
The absolute change frequency and absolute deviation trend of carbon trading prices with B under the characteristic time scale. (a1) China, (a2) Europe. (b1) China, (b2) Europe. (c1) China, (c2) Europe. (d1) China and (d2) Europe.

**Fig 3:**
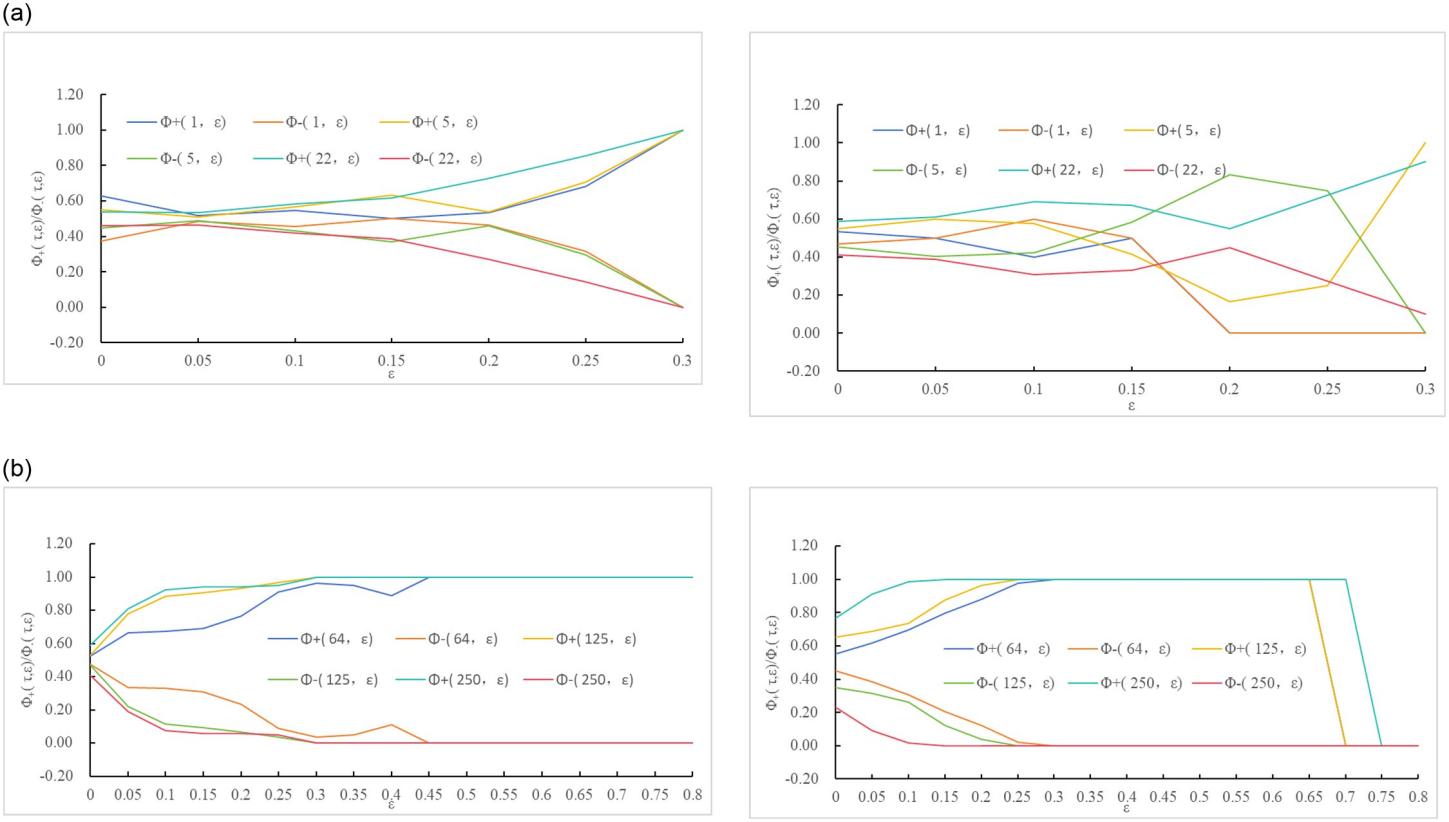
The relative frequency of carbon trading price changes with B under the characteristic time scale. (a1) China, (a2) Europe. (b1) China and (b2) Europe.

Utilizing Eqs (3), (4) and (5), the absolute and relative change frequencies, along with the absolute deviations, were calculated for different ε values (0 ≤ ε ≤ 1.6, h(Step length) = 0.05) at characteristic time scales. The results are shown in Figs 2 and 3.

As shown in Figs 2 and 3, investors’ expected returns (ε) exert a certain impact on the carbon trading prices in China and Europe, leading to price distortions. However, these distortions are not indefinite. As ε changes, both the absolute and relative change frequencies gradually reach saturation (as shown in Table 1) and cease to change. This can be attributed to the volatility of carbon trading prices in China and Europe not meeting investors’ excessively high expected returns. Therefore, for investors seeking profit, adjusting their psychological expectations of returns becomes essential.

**Table 1:** The various frequencies of carbon trading prices converge to the saturation critical point εc(τ) under the characteristic time scale.

εc(τ)	τ = 1	τ = 5	τ = 22	τ = 64	τ = 125	τ = 250
Panel A: China						
p+(τ, ε)	0.40	0.40	0.60	0.95	0.95	0.95
p0(τ, ε)	0.40	0.40	0.60	0.95	0.95	0.95
p-(τ, ε)	0.30	0.30	0.30	0.45	0.30	0.30
Φ+(τ, ε)	0.30	0.30	0.30	0.45	0.30	0.30
Φ-(τ, ε)	0.30	0.30	0.30	0.45	0.30	0.30
Panel B: Europe						
p+(τ, ε)	0.20	0.35	0.50	0.70	0.70	0.75
p0(τ, ε)	0.20	0.35	0.50	0.70	0.70	0.75
p-(τ, ε)	0.20	0.30	0.40	0.30	0.25	0.15
Φ+(τ, ε)	0.20	0.30	0.40	0.30	0.25	0.15
Φ-(τ, ε)	0.20	0.30	0.40	0.30	0.25	0.15

Based on the analysis of Fig 2, the carbon trading price volatility behavior under different ε values in China and Europe can be summarized as follows:

1. The higher the ε value, the more extreme the judgments on the price fluctuations in China and Europe tend to be (as shown in Fig 2a1, 2a2, 2c1 and 2c2). For example, when τ = 1, ε < 0.4 suggests that the carbon trading prices in China are random, and ε ≥ 0.4 indicates a probability of 0 for price fluctuations. When τ = 1, ε < 0.2 suggests that the carbon trading prices in Europe are random, and ε ≥ 0.2 indicates a probability of 0 for price fluctuations.
2. Under various characteristic time scales, as ε increases, the absolute deviations of carbon trading price fluctuations in China and Europe generally exhibit a trend of initially increasing, then decreasing, and eventually reaching saturation.
3. The price fluctuations of carbon trading in China and Europe are asymmetric. Before reaching saturation, the absolute deviations are not 0, but the probability of upward movements is generally larger than that of downward movements. For example, when τ = 22 and ε = 0.15, the probability of upward movements in the Chinese carbon market is 3.1% higher than the probability of downward movements (as shown in Fig 2d1); in the European carbon market, the probability of upward movements is 6.50% higher than the probability of downward movements (as shown in Fig 2d2).

Based on Fig 3, we can analyze the relative change frequencies of carbon trading prices in China and Europe from the perspective of trading time intervals.

1. For investors with short trading time intervals (τ = 1,5,22), when ε < 0.15, the probability of price fluctuations in Chinese and European carbon trading prices is similar, and the trends are relatively close, exhibiting moderate fluctuations. This suggests that the behavior of carbon trading prices in China and Europe is closer to a random walk. Investors in this scenario focus on short to medium-term growth and are willing to take on small investment risks that they can afford while maintaining their existing capital. However, when ε ≥ 0.15, the relative change frequency fluctuates significantly and quickly converges to extreme values of 1 or 0 (as shown in Fig 3a).
2. For investors with long trading time intervals (*τ* = 64,125,250), the probability of price fluctuations in the Chinese and European carbon markets differs significantly. Chinese investors, represented by ε = 0.05, and European investors, represented by ε = 0.1, exhibit a fast-changing trend that fluctuates rapidly and saturates (as shown in Fig 3b). In this case, investors are more focused on long-term and significant capital appreciation. They are not in urgent need of immediate returns and have a high tolerance for fluctuation in returns.

From Table 1, several observations can be made:

1. Under the same characteristic time scale τ, the convergent saturation points of various frequencies for European carbon trading prices are generally smaller than those for China. This indicates that, at the same trading time intervals, investors in the Chinese carbon trading market exhibit a greater inclination for higher returns compared to their European counterparts. The relatively new and opportunistic nature of China’s carbon market attracts investors seeking substantial profitability. In contrast, the mature and established European carbon trading market leads investors to adopt a more cautious approach, resulting in smaller saturation points compared to China.
2. The convergent saturation points of *p*_+_(*τ*, *ε*), *p*_0_(*τ*, *ε*), *p*_−_(*τ*, *ε*) and *ϕ*_+_(*τ*, *ε*), *ϕ*_−_ (*τ*, *ε*) remain the same across different characteristic time scales.
3. When *p*_+_(*τ*, *ε*) and *p*_0_(*τ*, *ε*) occur in the carbon trading markets of China and Europe, the longer the characteristic time scale τ, the higher the saturation point *ε*_*c*_(*τ*) tends to be. This indicates that, when the absolute change frequency of carbon trading prices remains constant or increasing, long-term investors in the carbon trading markets of China and Europe have higher expectations of returns compared to short-term investors.

### The absolute change frequency and absolute deviation of carbon trading prices in China and Europe

Using Eqs (3) and (5), the absolute change frequency (*p*_+_(*τ*, *ε*), *p*_0_(*τ*, *ε*), *p*_−_(*τ*, *ε*)) and absolute deviation (*d*_*a*_(*τ*, *ε*)) of carbon trading prices in China and Europe under different τ and ε values can be obtained *p*_+_(*τ*, *ε*), *p*_0_(*τ*, *ε*), *p*_−_(*τ*, *ε*).

**Fig 4:**
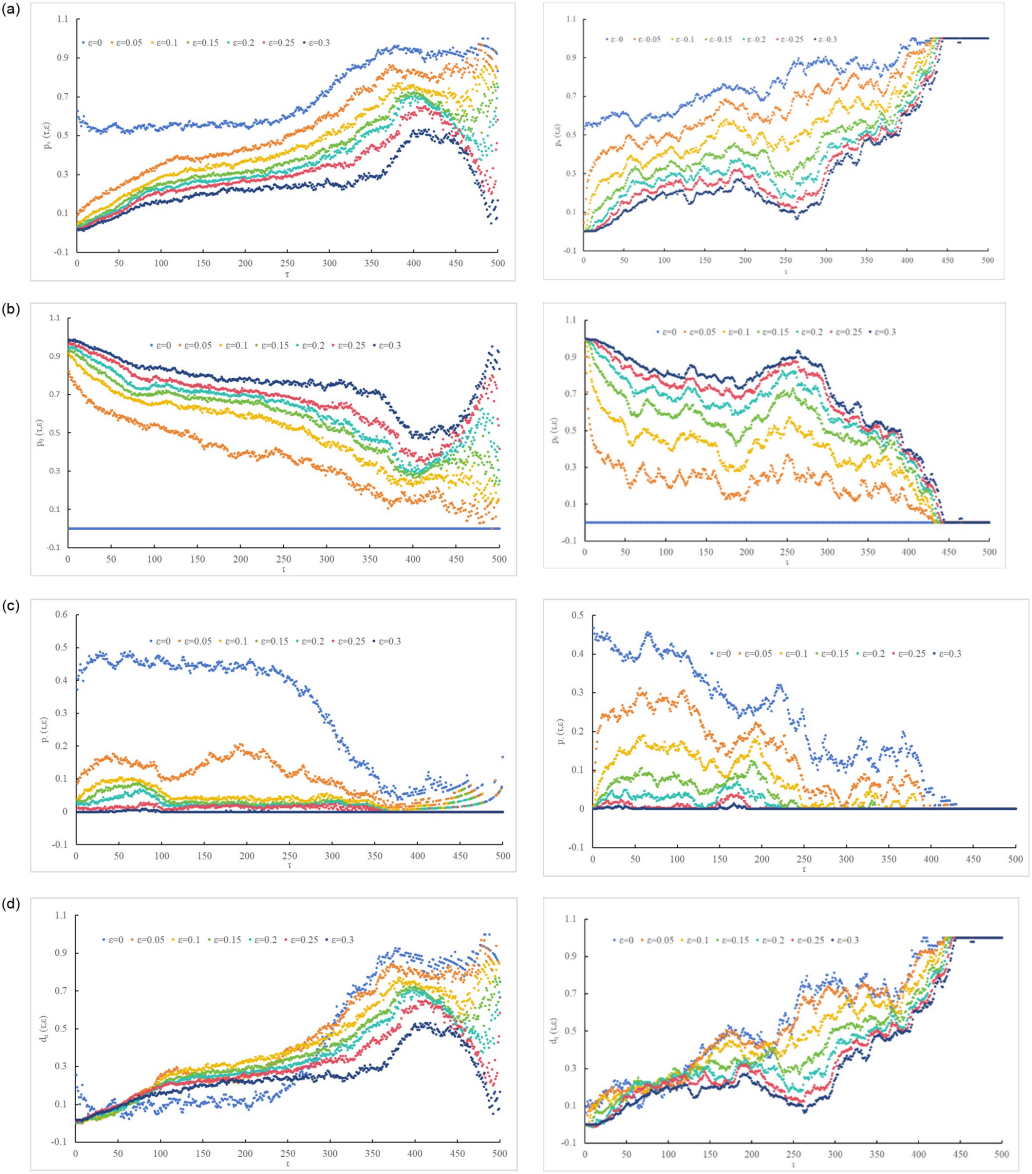
Investors’ perception of carbon trading price information (absolute frequency of change and absolute deviation). (a1) China, (a2) Europe. (b1) China, (b2) Europe. (c1) China, (c2) Europe. (d1) China, (d2) Europe.

Fig 4 illustrates the changing trends of the absolute change frequency and absolute deviation of carbon trading prices in China and Europe under different τ values, using ε = 0.05,0.1,0.15,0.2,0.25,0.3 (with ε = 0 as a reference). The figure visually depicts the distortion between investors’ perceptions and the actual carbon trading price behavior, as detailed in Table 2.

**Table 2:** Information distortion of carbon trading investors’ *ε* reference *p*_+_(*τ*, 0), *p*_0_(*τ*, 0) and *p*_−_(*τ*, 0) under the characteristic time scale.

εc(τ)	τ = 1	τ = 5	τ = 22	τ = 64	τ = 125	τ = 250
Panel A: China						
p+ (τ, ε)-p+ (τ, 0)						
ε = 0.05	-0.54	-0.44	-0.36	-0.25	-0.15	-0.08
ε = 0.1	-0.58	-0.49	-0.43	-0.32	-0.21	-0.18
ε = 0.15	-0.59	-0.50	-0.46	-0.34	-0.26	-0.23
ε = 0.2	-0.60	-0.52	-0.47	-0.36	-0.28	-0.28
ε = 0.25	-0.60	-0.53	-0.49	-0.39	-0.31	-0.30
ε = 0.3	-0.61	-0.53	-0.49	-0.40	-0.36	-0.36
p- (τ, ε)-p- (τ, 0)						
ε = 0.05	-0.29	-0.35	-0.30	-0.34	-0.36	-0.29
ε = 0.1	-0.33	-0.41	-0.38	-0.38	-0.43	-0.37
ε = 0.15	-0.34	-0.42	-0.41	-0.40	-0.44	-0.39
ε = 0.2	-0.35	-0.43	-0.43	-0.42	-0.45	-0.39
ε = 0.25	-0.36	-0.44	-0.45	-0.46	-0.46	-0.39
ε = 0.3	-0.37	-0.45	-0.46	-0.47	-0.47	-0.41
p0 (τ, ε)-p0 (τ, 0)						
ε = 0.05	0.82	0.79	0.66	0.59	0.51	0.37
ε = 0.1	0.91	0.90	0.81	0.69	0.64	0.55
ε = 0.15	0.93	0.93	0.87	0.74	0.70	0.61
ε = 0.2	0.95	0.95	0.90	0.78	0.73	0.67
ε = 0.25	0.96	0.97	0.93	0.85	0.77	0.69
ε = 0.3	0.98	0.98	0.96	0.87	0.83	0.77
Panel B: Europe						
p+ (τ, ε)-p+ (τ, 0)						
ε = 0.05	-0.49	-0.29	-0.19	-0.09	-0.11	-0.19
ε = 0.1	-0.53	-0.49	-0.33	-0.18	-0.25	-0.32
ε = 0.15	-0.53	-0.54	-0.46	-0.21	-0.34	-0.47
ε = 0.2	-0.53	-0.55	-0.53	-0.29	-0.38	-0.56
ε = 0.25	-0.53	-0.55	-0.57	-0.36	-0.43	-0.63
ε = 0.3	-0.53	-0.55	-0.57	-0.42	-0.47	-0.66
p- (τ, ε)-p- (τ, 0)						
ε = 0.05	-0.42	-0.28	-0.16	-0.16	-0.10	-0.18
ε = 0.1	-0.46	-0.41	-0.30	-0.29	-0.20	-0.23
ε = 0.15	-0.47	-0.44	-0.35	-0.36	-0.30	-0.23
ε = 0.2	-0.47	-0.44	-0.37	-0.41	-0.34	-0.23
ε = 0.25	-0.47	-0.45	-0.40	-0.44	-0.35	-0.23
ε = 0.3	-0.47	-0.45	-0.41	-0.45	-0.35	-0.23
p0 (τ, ε)-p0 (τ, 0)						
ε = 0.05	0.91	0.57	0.34	0.25	0.21	0.37
ε = 0.1	0.99	0.90	0.62	0.46	0.45	0.55
ε = 0.15	1.00	0.98	0.81	0.58	0.64	0.71
ε = 0.2	1.00	0.99	0.90	0.71	0.72	0.80
ε = 0.25	1.00	0.99	0.96	0.80	0.78	0.86
ε = 0.3	1.00	1.00	0.98	0.87	0.81	0.89

Table 2 reveals that the expected return ε generates negative distortion for *p*_+_(*τ*, *ε*), *p*_−_(*τ*, *ε*) and positive distortion for *p*_0_(*τ*, 0), with the distortion being most pronounced for *p*_0_(*τ*, 0). Additionally, as the trading time interval τ increases, the distortion caused by ε generally tends to decrease.

Fig 4 illustrates the self-similarity and intrinsic consistency in investors’ perceptions with different expectations (*ε* > 0) regarding the rise, stability, and decline of carbon trading prices in China and Europe. Examining the evolutionary paths of *p*_+_(*τ*, *ε*), *p*_0_(*τ*, *ε*) and *p*_−_(*τ*, *ε*), it can be observed that the carbon markets in China and Europe exhibit different developmental trends. The distortion behavior in the Chinese carbon market tends to approach 1 and shows a scattered downward trend as τ increases. On the other hand, the distortion behavior in the European carbon market gradually weakens with the increase of τ and does not significantly impact the long-term trend of prices.

In the Chinese carbon market, investors have a short-term and unstable bullish perception of carbon trading prices. With the increase of τ, *p*_+_(*τ*, *ε*) and *d*_*a*_(*τ*, *ε*) exhibit a non-convergent trend of initial growth followed by a decrease. In contrast, investors in the European carbon market have a long-term and stable bullish perception of carbon trading prices. As τ increases, *p*_+_(*τ*, *ε*) and *p*_−_(*τ*, *ε*) gradually converge to 1, while *p*_0_(*τ*, *ε*) and *d*_*a*_(*τ*, *ε*) gradually converge to 0.

1. When ε ≤ 0.15 occurs, the divergence in investors’ perception of the European carbon trading market is mainly driven by the magnitude and timing of price fluctuations rather than the fluctuations themselves. Their perception of *p*_±_(*τ*, *ε*) (or *p*_0_(*τ*, *ε*)) rapidly increases (or sharply decreases) within τ ≤ 56 (i.e., trading time intervals of less than one quarter) and then slowly increases (or decreases) in oscillations, resulting in significant volatility.
2. When ε > 0.15 occurs, the distortion between investors’ perception of European carbon trading price behavior and the actual price behavior is much greater compared to ε ≤ 0.15. Therefore, investment trading based on this perception would severely distort carbon trading price behavior, leading to drastic price fluctuations and exacerbating risk and uncertainty in the European carbon trading market.
3. When ε ≤ 0.15 occurs, investors have a bullish perception of European carbon trading prices within the time scale of 0 ≤ τ ≤ 436. If it exceeds 436, carbon trading price behavior is considered random, and both long and short positions may be profitable. When ε > 0.15 occurs, investors hold a long-term bullish perception of European carbon trading prices. With the increase of τ, almost all absolute deviations (i.e., *d*_*a*_(*τ*, *ε*)) become positive.

### The relative frequency of fluctuations in carbon trading prices between China and Europe

**Fig 5:**
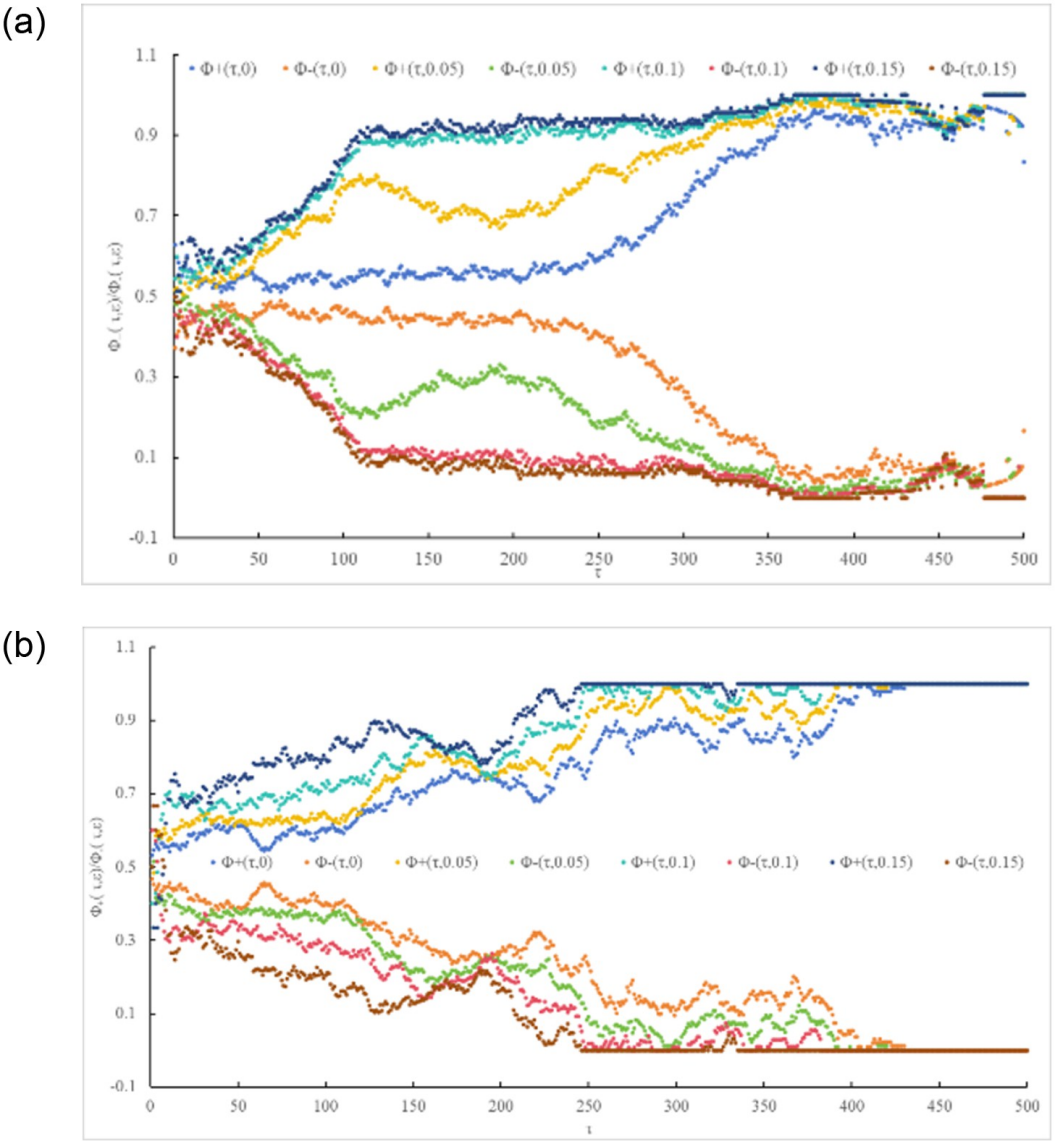
Investors’ perception of carbon trading price information①. (a) China and (b) Europe.

**Fig 6:**
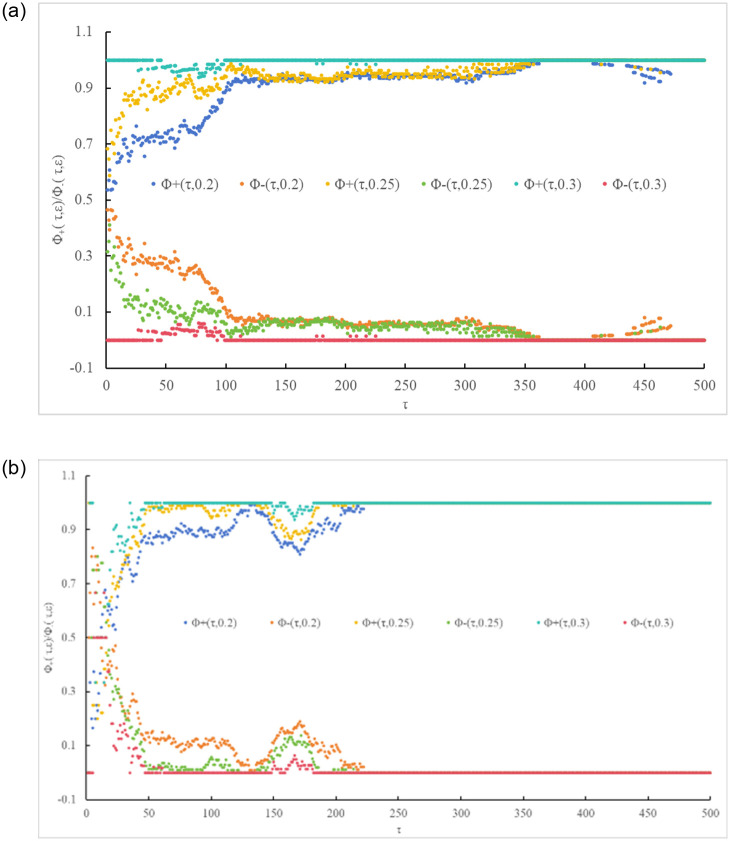
Investors’ perception of carbon trading price information②. (a) China, (b) Europe.

Although investors’ perception of the absolute unchanged frequency of carbon trading prices in China and Europe (i.e., *p*_0_(*τ*, *ε*)) results in the greatest distortion caused by ε, investors are more concerned about the occurrence of price changes. Therefore, it is necessary to analyze the situation of carbon trading price changes (i.e., relative change frequency). Based on Eq (4), the relative change frequencies (*ϕ*_±_(*τ*, *ε*)) of carbon trading prices in China and Europe are shown in Figs 5 and 6, respectively. Similarly, the distortion of actual carbon trading price information in China and Europe by ε is also studied in terms of relative change frequencies (see Table 3).

**Table 3:** The distortion of ε reference *ϕ*_+_ (*τ*, 0) information for carbon trading market investors under the characteristic time scale.

Φ+(τ,ε)	τ = 1	τ = 5	τ = 22	τ = 64	τ = 125	τ = 250
Panel A: China						
ε = 0.05	-0.11	-0.04	0.00	0.14	0.25	0.22
ε = 0.1	-0.08	0.02	0.04	0.15	0.11	0.33
ε = 0.15	-0.13	0.08	0.08	0.17	0.02	0.35
ε = 0.2	-0.09	-0.01	0.19	0.24	0.03	0.35
ε = 0.25	0.06	0.16	0.32	0.39	0.03	0.36
ε = 0.3	0.37	0.45	0.46	0.44	0.03	0.41
Panel B: Europe						
ε = 0.05	-0.03	0.05	0.02	0.07	0.04	0.14
ε = 0.1	-0.13	0.03	0.10	0.14	0.05	0.22
ε = 0.15	-0.03	-0.13	0.08	0.24	0.14	0.23
ε = 0.2	-0.53	-0.38	-0.04	0.33	0.09	0.23
ε = 0.25	-0.53	-0.30	0.14	0.43	0.04	0.23
ε = 0.3	-0.53	0.45	0.31	0.45	0.00	0.23

The results indicate that, unlike the absolute change frequency, the distortion caused by ε under the relative change frequency (*ϕ*_+_(*τ*, 0)) is mostly positive, with negative distortion occurring only at shorter and longer time scales (τ = 1 and τ = 5 in the Chinese market; τ = 1, τ = 5 and τ = 22). Similarly, this distortion generally tends to decrease as the time scale (τ) increases. This implies that the distortion of carbon trading price behavior by ε, whether in terms of absolute or relative change frequency, does not affect the long-term trend of prices. As shown in Figs 5 and 6, variations in the perception of historical carbon trading price information (*ϕ*_±_(*τ*, 0)) in China and Europe among investors are evident in terms of relative change frequency. Overall, their perception is similar to *ϕ*_±_(*τ*, 0) in the former case and differs from *ϕ*_±_(*τ*, 0) in the latter case.

1. For the Chinese market, when the expected returns are low, the changes in carbon trading prices exhibit an ordered pattern. *ϕ*_+_(*τ*, *ε*) is mostly above 0.5, while *ϕ*_−_(*τ*, *ε*) is mostly below 0.5. As the time scale (τ) extends, *ϕ*_+_(*τ*, *ε*) significantly increases until saturation (see Figs 5(a) and 6(b)). This indicates that there is a positive trend in carbon trading price behavior in China, rather than being unbiased, implying a higher probability of long-term bullishness. This result is consistent with the analysis of absolute change frequency and absolute deviation in the previous analysis.
2. For the European market, when the expected returns are low, the changes in carbon trading prices are comparatively distinct. Similar to the Chinese market, *ϕ*_+_(*τ*, *ε*) is mostly above 0.5, while *ϕ*_−_(*τ*, *ε*) is mostly below 0.5. As the time scale (τ) extends, *ϕ*_+_(*τ*, *ε*) significantly increases, reaching saturation in the case of China’s *τ* = 365 and Europe’s *τ* = 99 (see Figs 5(b) and 6(b)).

## Conclusion

1. The carbon trading prices in China and Europe exhibit asymmetric behavior. When τ < 277, the *d*_*a*_ value changes gradually but Chinese market still indicates a bullish trend. When τ ≥ 277, the *d*_*a*_ value increases rapidly, indicating a clear bullish sentiment among investors towards carbon trading prices in China. Despite variations in both τ and ε, the *d*_*a*_ value consistently demonstrates a linear upward trend, reflecting a significant overall bullish outlook in the European market. This suggests a higher probability of long-term bullishness in carbon trading prices.
2. Investors’ choices of investment time scale (τ) and expected returns (ε) exert significant influence on the behavior of carbon trading prices in both China and Europe. A longer τ implies a higher probability of bullish trends in carbon trading prices for both regions. As for ε, a higher value leads to more extreme judgments regarding the fluctuations in carbon trading prices, resulting in increased distortion in the behavior in China and Europe. Investors with an investment time scale of less than one month anticipate volatility in carbon trading prices in both China and Europe. Specifically, when ε < 0.15, there is little difference in the probability of price increase and decrease, suggesting random behavior in carbon trading prices. Conversely, when ε ≥ 0.15, the relative change frequency fluctuates significantly, rapidly converging to extreme values of 1 or 0. On the other hand, investors with an investment time scale exceeding one quarter adopt a more tempered perspective. They exhibit a long-term bullish outlook on carbon trading prices in both China and Europe. Moreover, with an increase in ε, the probability of bullishness fluctuates rapidly until reaching a saturation point. Once saturation is achieved, there is no further distortion in the behavior of carbon trading prices in both regions.
3. In the Chinese carbon trading market, a discernible positive trend characterizes the behavior of carbon trading prices, indicating a higher probability of long-term bullishness. Conversely, in the European market, when expected returns are low, carbon trading prices exhibit more pronounced volatility, thereby exacerbating the risk and uncertainty in the European carbon trading market.

Existing research on carbon markets focuses on the trends in carbon price volatility, carbon market risks, market efficiency, and market construction. This paper explores the behavior of carbon market price volatility from the perspective of investor trading intervals and psychological expected returns, representing a micro-level investigation of individual behaviors within the carbon market. This approach allows for tailored policy formulation by the government to address the diverse needs of different investor types. However, delving into the underlying mechanisms and developing strategies to prevent and mitigate carbon market price volatility and associated risks from investor behavior and psychology necessitates further investigation in subsequent studies.
